# Development of quality indicators for non-small cell lung cancer care: a first step toward assessing and improving quality of cancer care in China

**DOI:** 10.1186/s12885-017-3602-0

**Published:** 2017-08-31

**Authors:** Xinyu Wang, Shaofei Su, Shouyi Li, Han Bao, Meiqi Zhang, Dan Liu, Hao Jiang, Jiaying Wang, Meina Liu

**Affiliations:** 1 Department of Biostatistics, Public Health College, Harbin Medical University, 157 Baojian Road, Harbin, 150081 Heilongjiang People’s Republic of China; 2 People’s Hospital of Jilin Province, Changchun, Jilin, People’s Republic of China

**Keywords:** Quality indicators, Quality of care, Lung cancer, Chinese health system

## Abstract

**Background:**

Large gap exists between clinical practice and recommended care and large room exists for the improvement of care quality for non-small cell lung cancer (NSCLC) in China. Results of some studies have shown that assessment of care quality can help to make improvement and the development of quality indicators is deemed as the initial and most essential part. Yet there is no such an indicators system specifically suitable for Chinese health care system. The goal of the study is to set up a group of Chinese quality indicators for NSCLC care and make it the first step towards the improvement of NSCLC care quality in China.

**Methods:**

We **c**onstructed a new indicator framework based on the characteristics of NSCLC care and the nature of Chinese health care system. Under the new framework, potential indicators were collected and a 3-round modified Delphi process was conducted by a national multi-disciplinary Expert Panel to develop a set of indicators until they reached the final consensus.

**Results:**

A new indicator framework (structure, process, communication, management of symptoms or treatment toxicity and outcome) was developed. Seventy four indicators were extracted from guidelines and relevant literatures as potential indicators; 43 indicators plus 1 suggested indicator were remained after the discussion of Round 1; questionnaires of Round 2 were rated by Expert Panel and 19 indicators met the inclusion criteria and entered Round 3; 2 of the eliminated indicators in Round 2 were retrieved by the Expert Panel at the in-person meeting (Round 3). Therefore, 21 indicators got the final consensus of the Expert Panel.

**Conclusions:**

Guided by the new indicator structure, a set of indicators suitable for Chinese healthcare system was developed and can be utilized to measure and improve the care quality of non-small cell lung cancer.

**Electronic supplementary material:**

The online version of this article (10.1186/s12885-017-3602-0) contains supplementary material, which is available to authorized users.

## Background

Lung cancer is the leading cause of cancer death all over the world, which is reported continuously as having the highest mortality rate [1–3]. Two main categories exist for lung cancers: small cell lung cancer, which accounts for 15% of the cases, and Non-small cell lung cancer (NSCLC), which accounts for the other 85% [4]. In past decades, significantly novel advances in diagnosis and treatment of NSCLC have been made and their effectiveness was supported by strong clinical evidence [5, 6]. Thereafter, clinical practice guidelines incorporating the latest medical advances for cancer care were updated and issued every year in China to guide the practice for NSCLC patients. However, studies showed that a slight increase, instead of an evident drop, could be seen in the mortality rate of lung cancers from 2002 to 2011 in China [7], which cast a doubt on whether more advanced guidelines could lead to better quality of care.

Quality of care (QOC) is defined as the degree to which health services for individuals and populations increase the likelihood of desired health outcomes and are consistent with current professional knowledge [8, 9]. As stated by several studies, a wide gap between actual practice and clinical practice guidelines was observed in quality of care for many diseases including NSCLC [10–14]. For example, it has been reported that many patients with early-stage NSCLC do not undergo surgery or adjuvant chemotherapy, which is suggested by most guidelines of NSCLC [15, 16]. It is also reported that reducing the gap between best evidence and clinical practice is associated with reductions in patient morbidity and mortality [11, 12, 17–19], and reduced healthcare costs [20]. To bridge the gap, current QOC must be assessed and efforts should be made based on the observations from the results of assessment. In 1999, the institute of medicine of USA issued a landmark report which called for attention to quality of cancer care in USA, and subsequently recommended consecutive steps to improve quality of cancer care, among which development of quality indicators was recognized to be the essential and first step for quality improvement [21]. Quality indicators are measurement tools of practice performance, for which there is evidence or consensus that they can be used to assess QOC of a particular health care process [22, 23]. Many countries such as America, Canada and Netherlands have already taken actions to establish multi-dimensional quality indicators to assess QOC in areas like breast cancer, colorectal cancer as well as lung cancer and most of them witnessed a remarkable improvement of care quality [24–26].

In China, concerning quality measurement for cancer care are indicators like concordance rate of admitting and discharging diagnosis and readmission rate, which can only assess limited process of cancer care. Considering the complex nature of NSCLC and the characteristics of Chinese healthcare system and referring to the results of other similar studies, we intend to set up a more comprehensive framework of indicators. The new framework should be able to assess aspects QOC as detailed and comprehensive as possible, which could help us get deeper insight into the current QOC. Based on such a framework, we can discover the specific drawbacks during the care of NSCLC and light up a direction for quality improvement. Moreover, due to the similar complexity of all cancers, the new framework is expected to act as a reference for other cancer assessment programs to validate its usefulness not only in china but also in other countries around the world.

The main goal of this study is to establish a new indicator framework for NSCLC care based on the classic structure-process-outcome framework and systematically develop a set of quality indicators specifically suitable for China using a modified Delphi process. The resulting set of indicators would serve as standard tools for measuring and monitoring quality of NSCLC care and act as guidance for quality improvement.

## Methods

### Panel selection

Panelists were selected from a variety of disciplines in order to reflect the multidisciplinary nature of NSCLC care. Nominations for members to the expert panel were requested from provincial professional organization. The Expert Panel consists of 16 members of whom 10 are medical oncologists, 5 are surgical oncologists, and 1 is radiation oncologist. The Panel has a broad geographic distribution including Beijing, Harbin, and Shanghai, representing the middle, north and south of China, respectively. Each of the panelists is authority in his or her area of expertise and all of them have clinical practice experience for more than 10 years. Furthermore, 12 of the 16 panelists are members of Chinese Anti-Cancer Association which represents the first class of knowledge and medical technique in cancer care.

### Generation of new indicator framework

The classical “structure-process-outcome” framework is often used in indicator development studies. Structure indicators describe the innate characteristics of healthcare providers such as the qualification and technique of them and the allocation of medical equipment [27]. While process indicators cover the procedures or methods of care delivery from diagnosis, treatment to follow-up, which will definitely reflect the QOC if properly chosen [28]. However, due to the complexity of NSCLC itself, the multifarious process of care, and the poor prognosis of NSCLC, we consider that more attention on communication between patients and doctors may play an important part in getting better outcome. Since proper communication can increase the satisfaction degree of patients thus can improve the compliance of patients to the prescription and treatment decisions of doctors; moreover, as lung cancer is often accompanied with pain, fatigue, depression, and other diseases caused by treatment, which often leads to inferior life quality even undesirable outcome of patients after discharging, we consider that a field relating to management of symptoms or treatment toxicity should exist between process and outcome.

Therefore, a new indicator framework including structure, process, communication, management of symptoms or treatment toxicity and outcome was built to guide the development of NSCLC indicators for care quality.

### Generation of potential indicators

Under the guidance of the new indicator framework, National Comprehensive Cancer Network (NCCN) clinical practice guideline [29] and Chinese clinical practice guideline for NSCLC were reviewed to extract recommendations in diagnosis and treatment as candidate quality indicators. A systematic literature search was also conducted in electronic databases using searching terms “lung cancer”, “quality indicator”, “quality of care”, “quality assessment”, and “performance measure”. Quality indicators for assessment in the area of NSCLC developed in other countries were also included in this study as candidate indicators (All the candidate indicators and the reference studies in this part are shown in Additional file 1: Table S1). Potential indicators were classified into 5 domains under the framework. Their English and Chinese names with detailed definition were prepared to be discussed in the first round.

### Delphi process

#### Round 1-Preliminary screening of indicators

One radian oncologist, two surgical oncologists, and one internal oncologist from Expert Panel were invited to discuss the potential indicators. During the discussion, experts focused on the definitions and data availability of each indicator as well as similarity among indicators. Modifications, eliminations, and combinations were made based on the above considerations and experts were encouraged to add additional indicators into the list based on their experience. Therefore, a shortened list of indicators was created.

#### Round 2- Rating of indicators

The indicators confirmed in the first round were formulated into a Delphi questionnaire with a letter introducing the background and the aim of the study as well as detailed instructions of six rating criteria for each indicator: evidence-basis, usefulness, interpretability, validity, preventability, and the feasibility of data collection. The rating scale of each indicator was a five-point Likert scale (see Table 1). The questionnaire was distributed by e-mail to the 16 expert panel members, followed by a reminder e-mail 2 weeks later.

**Table 1:** Example of Delphi questionnaire

Title of indicator:						
Definition:						
Criteria	Totally disagree	Moderately agree			Totally agree	Score
1. Scientific evidence (The scientific evidence is sufficient)	1	2	3	4	5	
2. Usefulness (The indicator is capable of being guidance of clinical practices)	1	2	3	4	5	
3. Interpretability (The indicator can be interpreted by clinicians)	1	2	3	4	5	
4. Validity (The indicator can measure the quality of care and has potential for improvement in clinical practices)	1	2	3	4	5	
5. Preventability (The indicator has ability of prevent adverse outcomes)	1	2	3	4	5	
6. Feasibility (The feasibility of data collection)	1	2	3	4	5	
Overall Assessment	Cannot include	Could include			Must include	Score
	1	2	3	4	5	
Suggestions:						

For each of the 6 criteria and the overall assessment of each indicator, the inclusion criteria is: ① the mean score is equal to or greater than 4; ② the coefficient of variation is equal to or less than 0.25; ③ at least 13 of 16 (81.25%) experts rated the criteria equal to or greater than 4.

#### Round 3- Face-to-face meeting

Six experts and two biostatisticians as well as three research leaders attended the meeting which was held in Harbin in October, 2013. Experts were asked to freely discuss the rating result of each indicator; besides, whether the indicator was suitable for the measurement of NSLCL care in the environment of China health care system was also discussed at the face-to-face meeting; moreover, the eliminated indicators in Round 2 were reviewed again to decide whether some of them were also important and could be retrieved. The confirmation of inclusion and exclusion criteria for patients of each indicator was another important target of the meeting. After that, the research leaders and biostatisticians discussed the whole study design including questionnaire for data collection, the sample size, way of indicators reporting, and the statistical methods for assessing and comparing the quality of care for NSCLC among hospitals. The result of the meeting and the final set of indicators was made into a form with inclusion and exclusion criteria for patients in it and was then sent to the other panel members who could not make it to the meeting. Feedback was received 1 week later and no more disagreement was observed, which indicated the final set of indicators received clear consensus by panel experts and can be applied in the following steps of evaluating the quality of NSCLC care.

## Results

There was a total of 74 potential indicators that had been extracted from guidelines and literatures, of which 44 were for process, 9 for management of side-effects, 7 for structure, communication, and outcome, respectively (see Additional file 1: Table S1). All these indicators were made into a Delphi questionnaire to be discussed in Round 1.

### Round 1

In the first round, 31 indicators were either excluded for lacking data availability (such as psychosocial problems consultation) or merged for having similar definitions (such as two indicators concerning multidisciplinary team discussion). Some indicators such as “FEV1 and DLCO obtained before pulmonary resection” and “ECG obtained before pulmonary resection” were restricted with time length of “within 2 calendar weeks”. Besides, “proportion of NSCLC patients staging IIIB or IV who receive imaging study to assess response of chemotherapy at least once before the completion of four cycles” was newly suggested by experts. At the end of this round, 44 indicators were remained and made into a Delphi questionnaire (Table 1) to be rated by the Expert Panel.

### Round 2

The valid response rate of Delphi questionnaire in round 2 was 100%. According to the predefined inclusion criteria, 19 indicators met the criteria and finally enter the third round.

### Round 3

In this round, all the indicators which met the predefined criteria in Round 2 were remained and the indicator “EGFR test obtained before combination therapy” and the outcome indicator “the occurrence of postoperative complications” which were eliminated in Round 2 were retrieved by consensus from the Expert Panel because they were deemed important and necessary for quality measurement.

After completing all the procedures of Delphi approach, a total of 21 indicators including 1 structure indicator, 16 process indicators, 3 indicators for communication, and 1 outcome indicator were developed. The ratings of selected indicators are shown in Table 2 and the detailed indicator definition is listed in Additional file 1: Table S2.

**Table 2:** Summarized ratings of indicators retained from the rating round

Title.	Rating criteria and overall assessment (mean, coefficient of variation (%) and selectivity (%))						
	I-1	I-2	I-3	I-4	I-5	I-6	OVERALL
Structure indicators							
Availability of multidisciplinary lung cancer team	4.88 7.01 100.00	4.88 7.01100.00	4.81 8.38100.00	4.88 10.26 93.75	4.81 8.38100.00	4.56 13.79 93.75	4.75 9.42100.00
Process indicators							
Proportion of clinical stage III NSCLC patients for which a skeletal scintigraphy and a CT or MRI of the brain is done before the initiation of combination therapy	4.69 10.21 100.00	4.56 11.23100.00	4.44 14.18 93.75	4.50 18.14 93.75	4.44 14.18 93.75	4.56 13.79 93.75	4.50 14.05 93.75
Proportion of NSCLC patients in advanced stages who receive performance status assessment	4.81 11.30 93.75	4.50 19.88 87.50	4.62 15.54 87.50	4.38 21.88 81.25	4.69 12.84 93.75	4.44 16.39 87.50	4.50 16.23 87.50
Proportion of NSCLC patients who receive EGFR test before combination therapy	4.81 8.38100.00	4.62 13.39 93.75	4.56 11.23100.00	4.62 10.81100.00	4.06 26.16 75.00	4.38 24.86 87.50	4.56 13.79 93.75
Proportion of pathology report available in the chart for NSCLC patients who have surgical resection	4.81 11.30 93.75	4.81 8.38100.00	4.75 12.15 93.75	4.56 17.84 81.25	4.50 19.88 87.50	4.69 12.84 93.75	4.75 14.38 87.50
Proportion of NSCLC patients who obtain FEV1 and DLCO within 2 weeks before lung resection	4.75 9.42 100.00	4.62 13.39 93.75	4.69 10.21100.00	4.56 13.79 93.75	4.56 13.79 93.75	4.69 12.84 93.75	4.62 10.81100.00
Proportion of NSCLC patients who receive ECG within 2 weeks before lung resection	4.56 13.79 93.75	4.56 13.79 93.75	4.62 10.81100.00	4.44 16.39 87.50	4.44 20.10 87.50	4.56 15.94 87.50	4.50 16.23 87.50
Proportion of NSCLC patients staging I or II without contraindications who undergo curative resection	4.75 12.15 93.75	4.69 10.21100.00	4.75 12.15 93.75	4.75 9.42100.00	4.62 15.54 87.50	4.50 19.88 87.50	4.69 12.84 93.75
Proportion of NSCLC patients staging IA without contraindications who receive lobectomy	4.56 15.94 87.50	4.50 14.05 93.75	4.88 7.01100.00	4.69 10.21100.00	4.19 21.74 81.25	4.50 14.05 93.75	4.50 14.05 93.75
Proportion of NSCLC patients staging IB to II who receive lobectomy with adjuvant chemotherapy or lobectomy only	4.44 21.72 81.25	4.50 11.48100.00	4.56 11.23100.00	4.50 14.05 93.75	4.38 21.88 81.25	4.50 16.23 87.50	4.50 14.05 93.75
Proportion of NSCLC patients with stage IIA, IIB or ΙΙΙA who receive adjuvant chemotherapy after curative resection	4.62 13.39 93.75	4.56 13.79 93.75	4.56 11.23100.00	4.62 10.81100.00	4.38 18.43 81.25	4.56 13.79 93.75	4.44 14.18 93.75
Proportion of NSCLC patients with stage IIA, IIB or ΙΙΙA who receive cisplatin-based adjuvant chemotherapy within 3 to 4 weeks after undergoing curative resection	4.69 10.21 100.00	4.56 11.23100.00	4.50 14.05 93.75	4.44 14.18 93.75	4.31 18.39 81.25	4.75 9.42100.00	4.62 10.81100.00
Proportion of NSCLC patients staging ΙΙΙB with malignant effusion or Ις who receive first-line chemotherapy	4.88 7.01 100.00	4.81 11.30 93.75	4.75 9.42100.00	4.81 8.38100.00	4.56 15.94 87.50	4.81 8.38100.00	4.75 9.42100.00
Proportion of NSCLC patients staging ΙΙΙB or Ις who receive imaging study to assess response of chemotherapy at least once before the completion of four cycles	4.88 7.01 100.00	4.75 9.42100.00	4.81 8.38100.00	4.75 9.42100.00	4.50 18.14 81.25	4.56 17.84 93.75	4.81 8.38100.00
Proportion of NSCLC patients staging I or II pathologically who receive postoperative radiation therapy after incomplete surgical resection	4.69 12.84 93.75	4.62 13.39 93.75	4.56 13.79 93.75	4.56 13.79 93.75	4.50 16.23 87.50	4.69 12.84 93.75	4.56 13.79 93.75
Proportion of locally advanced NSCLC patients who receive neo-adjuvant chemotherapy	4.50 16.23 87.50	4.56 13.79 93.75	4.50 14.05 93.75	4.56 11.23100.00	4.38 18.43 81.25	4.56 15.94 87.50	4.44 16.39 87.50
Proportion of locally advanced NSCLC patients with performance status 0 or 1 who receive combination therapy	4.88 7.01 100.00	4.88 7.01100.00	4.81 8.38100.00	4.75 12.15 93.75	4.75 12.15 93.75	4.56 13.79 93.75	4.88 7.01100.00
Communication indicators							
Proportion of NSCLC patients who are informed of a follow-up plan at the time of discharge from hospital	4.88 10.26 93.75	4.88 7.01100.00	4.94 5.06100.00	4.88 7.01100.00	4.88 7.01100.00	4.69 15.02 87.50	4.88 10.26 93.75
Proportions of active smokers with NSCLC who have had smoking cessation counseling documented	4.75 21.05 93.75	4.44 24.64 87.50	4.56 22.59 93.75	4.62 13.39 93.75	4.38 21.88 81.25	4.50 16.23 87.50	4.56 17.84 93.75
Proportion of NSCLC patients staging IA who are recommend adjuvant chemotherapy after curative resection (lower score: better)	4.69 12.84 93.75	4.56 15.94 87.50	4.62 13.39 93.75	4.44 16.39 87.50	4.38 18.43 81.25	4.44 20.10 87.50	4.50 16.23 87.50
Outcome indicators							
Post-operative complications	4.00 28.87 68.75	4.31 18.39 81.25	4.38 18.43 81.25	4.12 23.21 75.00	4.00 27.39 68.75	4.31 20.25 75.00	4.00 24.15 68.75

*I-1* scientific evidence, *I-2* utility, *I-3* interpretability, *I-4* validity, *I-5* preventability, *I-6* data availability, *CT* computed tomography, *MRI* magnetic resonance imaging, *EGFR* epidermal growth factor receptor, *FEV1* forced expiratory volume in one second, *DLCO* diffusing capacity of the lungs for carbon monoxide, *ECG* electrocardiogram. For each indicator, the first row listed mean ratings of each criteria, the second row listed coefficient of variation (%) and the third row listed selectivity for ratings of each criteria (%)

## Discussion

As far as we know, this is the first study focusing on the development of quality indicators for NSCLC in the context of Chinese heath care system and it is also the first study building and using the new indicator framework, which should be further tested by similar studies in other countries for its validity. After three round of modified Delphi process, a set of 21 indicators was developed. This set of indicators are supposed to quantify and visualize the gap between clinical practice and evidence-based guidelines; help us get a deeper and more comprehensive understanding of the current situation of NSCLC care in China thus put forward a clear direction of improvement. Under the guidance of the improvement direction, we can make effective interventions to bridge the gap in order to get better quality of care for NSCLC. We can also use these indicators to discover disparities of NSCLC care quality among hospitals, which is anticipated helpful to clinician, researchers, government administrators, and others who want to make decisions, policies, and changes based on the information.

Most previous studies developed indicators based on “structure-process-outcome” framework. There was a group from Netherlands who did it from professional, organizational, and patient-oriented perspectives and patient-oriented indicators made up almost half of the indicators [30]. This is a relatively new perspective of developing indicators. However, it is considered subjective and unreliable when using data from patients’ recall.

In this study, we pioneer the new indicator framework including five domains: structure, communication, process, management of symptoms or treatment toxicity, and outcome. The domain communication was built based on the consideration that good communication between doctors and patients plays an important role in quality improvement since patients tend to be more compliable to the treatment decision and prescription of doctors when they have better understanding of their illness thus making the process of care more smoothly. Some experts of other organizations also noticed the issue. In NCCN Oncology Policy Summit in 2013, panelists emphasized the importance of the communication between all doctors, nurses, and staff and patients as well as their families. They discussed how providing the “right” amount of information to patients and their families is a difficult task for physicians and nurses, but is critical to the patient experience. They also discussed how the overall culture of a hospital, or how patients and their families are received, all contribute to defining a quality experience [31]. As to the domain of management of symptoms or treatment toxicity, we consider that treatment side effects and toxicity are common in the process of cancer care, of which necessary management would have positive effect on prognosis and quality of life after discharging. In this study, four indicators related to this domain were selected in the first round of Delphi but all eliminated in the second round of rating. “The assessment of pain intensity” and “the reassessment of pain intensity” were excluded for not meeting any of the six criteria, suggesting that panelists did not think there were scientific evidence or the other five properties. The other two indicators “postoperative incentive spirometry” and “atrial fibrillation treated after lung resection within 45 minutes” were excluded because several experts thought that they lacked validity (the indicator can measure the quality of care and has potential for improvement) and preventability (the indicator has the ability of preventing adverse outcomes). Despite such a result, we still hold the point that the domain of “management of symptoms or treatment toxicity” is an important component of the proposed framework which aims to cover various aspects of care process. With the continuously updating guidelines, the indicators will be updated accordingly as well. The completeness of the framework also ensures that we follow the same methodology every time we renewal indicators. Experts from the Delphi process in this study may think the domain not as vital as others. However, the importance of this part for cancer care is undeniable. Another study of our team for cancer indicator development also validated the usefulness of this framework [32].

The Delphi process used in this study was consistent with previous studies [33–35]. However, some indicators developed in our study differed from those of others. Danish National Indicator project [36, 37] produced evidence-based indicators for eight diseases (including lung cancer) in 2000. The result included 9 indicators, all of which were outcome indicators. However, the result of this study had only one outcome indicator “postoperative complications”. Indicators presented in Danish study that did not pass rating in our project included “1-year survival rate” and “5-year survival rate”. The possible reasons are listed as followed: The first is that we put more emphasis on the comprehensiveness of indicators and the overall process of care in the current study; second, the follow-up information is inquired mainly by telephone in China. However, there is not yet a completed follow-up plan in all hospitals which means some hospitals have follow-up information while others do not and the register systems are not connected among hospitals; third, there is such a phenomenon in China that when patients are dead, their families are unwilling to tell strangers including doctors about the misfortune on the phone.

The result of the study includes 16 process indicators which cover four stages of NSCLC and almost every phase of care process including diagnosis, neo-adjuvant chemotherapy, surgery, adjuvant chemotherapy, radiotherapy, and documentation of pathology report. These process indicators are either evidence-based therapies or essential elements for appropriate treatment for NSCLC cancer patients and compliance to these indicators is supposed to improve the quality of care and decrease recurrence and mortality rate for patients.

The strengths of this study include a comprehensive review of evidence-based guidelines; a rigorous rating procedure that included criteria of scientific evidence, validity, interpretability, usefulness, preventability, and feasibility. The most unique feature that makes this study different from others is developing a new structure of indicators “structure, communication, process, management of symptoms or treatment toxicity, outcome”.

In the next step of the study, we will make a questionnaire to collect data from electronic medical records based on the final set of indicators and compute performance scores using appropriate statistical methods for each indicator of each hospital that are enrolled in this study. Feedback will be sent back to hospitals and doctors to help them make improvement strategies. The performance after feedback will be reassessed to examine the effect of intervention. We believe that aiming at the improvement of performance of selected indicators will lead to improved patient outcomes.

There are several limitations to this study. The first is that we only chose experts in lung cancer care because the process of developing indicators required a detailed understanding of the evidence base and clinical practice. Other perspectives like the ones of patients are also important because they are the receivers of care and their interests may vary from those of lung cancer experts; the second is that the indicators were determined by a group of experts, another group of experts with different discipline structure may rate the same potential indicators differently; the last limitation, which is also to be solved in our next step, is that the indicators should be up to date to reflect ever-changing medical progress in NSCLC and in Chinese healthcare system.

## Conclusions

NSCLC quality indicators developed in this study provide a firm foundation for future initiatives aimed at assessing and improving quality of care in China. The indicators differ from those of other organizations but are well suited to Chinese health care system and the indicator framework should be further addressed by other researchers to validate its usefulness.

## Additional file

Additional file 1: Table S1. Potential indicators extracted from guidelines and literatures. **Table S2.** Definition of the final 21 indicators. (DOCX 54 kb)

## Abbreviations

DLCO: Diffusion capacity of the lung for carbon monoxide
ECG: Electrocardiogram
EGFR: Epidermal growth factor receptor
FEV1: Forced expiratory volume in one second
NCCN: National Comprehensive Cancer Network
NSCLC: Non-small cell lung cancer
QOC: Quality of care
