# Supplementary material for: Development of quality indicators for non-small cell lung cancer care: a first step toward assessing and improving quality of cancer care in China
Source: BMC Cancer. 2017 Aug 31;17:603. doi: 10.1186/s12885-017-3602-0 (PMC5579936; doi:10.1186/s12885-017-3602-0)
Supplement: Additional file 1: Table S1. — Potential indicators extracted from guidelines and literatures. Table S2. Definition of the final 21 indicators. (DOCX 54 kb) [file 12885_2017_3602_MOESM1_ESM.docx]

**Table S1.** Potential indicators extracted from guidelines and literatures.

| **NO.** | **Indicator Title**^*^ |
| --- | --- |
|  | **Structure** |
| 1 | Thoracic surgery performed at high-volume hospital as opposed to low-volume hospital.[[1](#_ENREF_1)] |
| 2 | Thoracic surgery performed at National Cancer Institute designated cancer centers as opposed to other centers.[[2](#_ENREF_2)] |
| 3 | Thoracic surgery performed at a teaching facility as opposed to nonteaching facility.[[3](#_ENREF_3)] |
| 4 | Availability of treatment summary upon radiation treatment completion.[[4](#_ENREF_4)] |
| 5 | Availability of documented cancer stage.[[4](#_ENREF_4)] |
| 6 | Availability of a multidisciplinary lung cancer team. [[5-7](#_ENREF_5)] |
| 7 | Patients reported that they were informed about the existence of an oncology nurse specializing in lung cancer.[[6](#_ENREF_6), [7](#_ENREF_7)] |
|  | **Process** |
| 8 | Time from diagnosis to first treatment.[[8](#_ENREF_8)] |
| 9 | Patients started therapy within 35 calendar days from first visit to the pulmonologist.[[6](#_ENREF_6), [7](#_ENREF_7)] |
| 10 | Diagnostic trajectory completed within 21 calendar days from first visit to pulmonologist (chest CT scan, bronchoscopy, FDG-PET).[[5](#_ENREF_5)] |
| 11 | Availability of correlation with other relevant imaging studies (x-ray, CT scan, MRI) in diagnostic radionuclide scan report.[[9](#_ENREF_9)] |
| 12 | Documentation of at least one of the following mediastinal staging procedures in all patients with lung cancer:  Cervical mediastinoscopy  Positron emission tomography  Mediastinal lymphadenectomy.[[10](#_ENREF_10)] |
| 13 | Clinical stage III NSCLC for which a skeletal scintigraphy and a CT or MRI of the brain was done before the start of combination therapy.[[6](#_ENREF_6), [7](#_ENREF_7)] |
| 14 | For patients who had a surgical resection for stage I or II NSCLC, neoadjuvant chemotherapy was not to be given.[[11](#_ENREF_11)] |
| 15 | For patients who had a complete surgical resection and a pathological stage II or III NSCLC, adjuvant chemotherapy with a cisplatin-based regimen was used.[[11](#_ENREF_11)] |
| 16 | Stage IIIA and IIIB, neoadjuvant chemoradiation with resection or chemoradiation with platinum doublet.[[12](#_ENREF_12)] |
| 17 | Stage IIIA, pneumonectomy, lobectomy, or chemoradiation.[[13](#_ENREF_13)] |
| 18 | Patients assessed for family history of lung cancer.[[11](#_ENREF_11)] |
| 19 | Adjuvant chemotherapy received by patients after curative resection for NSCLC with stages IIA, IIB, or IIIA disease.[[14](#_ENREF_14), [15](#_ENREF_15)] |
| 20 | Adjuvant cisplatin-based chemotherapy received by patients within 60 days after curative resection for NSCLC with stages IIA, IIB, or IIIA disease.[[14](#_ENREF_14), [15](#_ENREF_15)] |
| 21 | Stage IV, chemotherapy.[[13](#_ENREF_13)] |
| 22 | Stage IV (including IIIB with malignant effusion), platinum doublet or single-agent chemotherapy[[12](#_ENREF_12)] |
| 23 | For patients with a stage IV or IIIB with malignant pleural effusion NSCLC and a performance status 2 at presentation, chemotherapy was given.[[11](#_ENREF_11)] |
| 24 | For patients with a stage IV or IIIB with malignant pleural effusion NSCLC who were elderly, but with good performance status and organ function, chemotherapy was given.[[11](#_ENREF_11)] |
| 25 | For patients with a stage IV or IIIB with malignant pleural effusion NSCLC who had a confirmed predominantly or entirely squamous cell carcinoma histology, bevacizumab was not used.[[11](#_ENREF_11)] |
| 26 | For patients with a stage IV or IIIB with malignant pleural effusion NSCLC with normal organ function and good performance status, first-line chemotherapy was a platinum-based doublet OR a third-generation agent doublet; bevacizumab may be added in selected cases.[[11](#_ENREF_11)] |
| 27 | For patients with a stage IV or IIIB with malignant pleural effusion NSCLC, first-line cytotoxic chemotherapy was not to be given beyond six cycles.[[11](#_ENREF_11)] |
| 28 | For patients with a stage IV or IIIB with malignant pleural effusion NSCLC who received second-line chemotherapy, the regimen was a single agent with either erlotinib, pemetrexed, or docetaxel.[[11](#_ENREF_11)] |
| 29 | Neoadjuvant chemotherapy for locally advanced NSCLC.[[16](#_ENREF_16), [17](#_ENREF_17)] |
| 30 | For patients who underwent concurrent chemoradiation, the concurrent chemotherapy regimen was platinum based.[[11](#_ENREF_11)] |
| 31 | Chemotherapy for advanced NSCLC.[[16](#_ENREF_16), [17](#_ENREF_17)] |
| 32 | Adjuvant radiation therapy recommended for patients after curative resection for NSCLC with stages IB or II disease (lower score-better).[[14](#_ENREF_14), [15](#_ENREF_15)] |
| 33 | For patients who had an incomplete surgical resection and a pathological stage I or II NSCLC, postoperative radiation therapy was given.[[11](#_ENREF_11)] |
| 34 | For patients who had a complete surgical resection and a pathological stage I or II NSCLC, adjuvant radiation therapy was not to be given.[[11](#_ENREF_11)] |
| 35 | Adjuvant radiation for stage I-III NSCLC.[[16](#_ENREF_16), [17](#_ENREF_17)] |
| 36 | Stage IIIB, chemoradiation.[[13](#_ENREF_13)] |
| 37 | Stage IIIB, with pleural effusion, chemotherapy with or without radiotherapy.[[13](#_ENREF_13)] |
| 38 | For patients with an unresected stage III NSCLC (except stage IIIB with malignant pleural effusion) and good performance status, concurrent chemoradiation was used as part of treatment.[[11](#_ENREF_11)] |
| 39 | Definitive chemoradiation for locally advanced NSCLC.[[16](#_ENREF_16), [17](#_ENREF_17)] |
| 40 | Locally advanced NSCLC with performance status 0 or 1 that was treated with combination therapy.[[6](#_ENREF_6), [7](#_ENREF_7)] |
| 41 | For patients who had surgery for NSCLC, a preoperative PET scan was performed.[[11](#_ENREF_11)] |
| 42 | FEV1 and DLCO obtained within 365 days before lung resection in all patients.[[10](#_ENREF_10)] |
| 43 | ECG obtained within 90 days before lung resection.[[10](#_ENREF_10)] |
| 44 | Documentation of at least one of the following after surgery in all patients:  1. Use of graduated elastic compression stockings  2. Use of lower extremity sequential compression devices  3. Administration of subcutaneous heparin[[10](#_ENREF_10)] |
| 45 | For patients with a clinical stage III or IV NSCLC, performance status was assessed. [[15](#_ENREF_15)] |
| 46 | Availability of pathology report after surgical resection.[[11](#_ENREF_11)] |
| 47 | For patients with a clinical stage I or II NSCLC at presentation and no medical contraindications, surgical resection for NSCLC was performed.[[11](#_ENREF_11)] |
| 48 | Stage IA, lobectomy.[[12](#_ENREF_12)] |
| 49 | Stage IB and II, lobectomy with adjuvant chemotherapy or lobectomy alone.[[12](#_ENREF_12)] |
| 50 | Patients received multidisciplinary consultation team discussion before treatment.[[11](#_ENREF_11)] |
| 51 | EGFR test obtained before combination therapy.[[18](#_ENREF_18), [19](#_ENREF_19)] |
|  | **Communication** |
| 52 | Patients reported that they were asked about psychosocial problems in family and problems at work.[[6](#_ENREF_6), [7](#_ENREF_7)] |
| 53 | Patients reported that they were asked about psychosocial stress factors and psychological symptoms.[[6](#_ENREF_6), [7](#_ENREF_7)] |
| 54 | Smoking history documented before lung resection.[[10](#_ENREF_10)] |
| 55 | Documented follow-up plan at time of patient discharge from hospital in all patients.[[10](#_ENREF_10)] |
| 56 | Documentation of smoking cessation consultation offered to all patients smoking at the time of or having quit within 30 days of pulmonary resection.[[10](#_ENREF_10)] |
| 57 | For patients who were active smokers, smoking cessation was advised and documented. |
| 58 | Adjuvant chemotherapy recommended and received after curative resection for NSCLC stage IA (lower score: better).[[15](#_ENREF_15)] |
|  | **Management of symptoms and treatment toxicity** |
| 59 | Patients reported that attention was paid to physical symptoms: pain, suffocation, nausea, fatigue, weight loss and insomnia.[[6](#_ENREF_6), [7](#_ENREF_7)] |
| 60 | Atrial fibrillation after lung resection treated within 45 minutes of its onset.[[10](#_ENREF_10)] |
| 61 | Documentation of postoperative incentive spirometry use in all patients.[[10](#_ENREF_10)] |
| 62 | After lung resection, for patients with analog pain of at least 6/10, treatment implemented with reassessment of pain score within 2 hours.[[10](#_ENREF_10)] |
| 63 | Treatment implemented with documented reassessment of pain score within 2 hours in all patients.[[10](#_ENREF_10)] |
| 64 | Documentation of analog pain scores≥6/10 during hospitalization in all patients.[[10](#_ENREF_10)] |
| 65 | Occurrence of significant pain at least 6/10 on analog scale.[[10](#_ENREF_10)] |
| 66 | Patients assessed with numeric pain scale at every episode of care.[[20](#_ENREF_20)] |
| 67 | Pain assessment documented.[[20](#_ENREF_20)] |
|  | **Outcome** |
| 68 | In-hospital and 30-day mortality after lung surgery.[[10](#_ENREF_10)] |
| 69 | Post-operative complication rate.[[11](#_ENREF_11)] |
| 70 | one-year survival rate.[[21](#_ENREF_21)] |
| 71 | Five-year survival rate.[[21](#_ENREF_21)] |
| 72 | Percentage of surgery patients dying within 30 days of surgery.[[20](#_ENREF_20)] |
| 73 | For surgical patient, 30-day in-hospital mortality rate.[[22](#_ENREF_22)] |
| 74 | For surgical patient, 30-day fatality rate.[[22](#_ENREF_22)] |

* The expression of the titles of 74 potential indicators may be different from those of the final selected indicators due to necessary modification and unification. The meaning of indicators remained unchanged.

**Table S2.** Definition of indicators

| **Title of indicator** | Numerator | Denominator |
| --- | --- | --- |
| **Structure indicators** |  |  |
| Availability of multidisciplinary lung cancer team |  |  |
| **Process indicators** |  |  |
| Proportion of clinical stage III NSCLC patients for which a skeletal scintigraphy and a CT or MRI of the brain is done before the initiation of combination therapy | NSCLC Patients for which a skeletal scintigraphy and a CT or MRI of the brain is done before the initiation of combination therapy | NSCLC patients Clinically staging III who accept treatment including radiotherapy， chemotherapy, surgery and so on |
| Proportion of NSCLC patients in advanced stages who receive performance status assessment | NSCLC patients who receive performance status assessment | NSCLC patients staging III or Ⅳ |
| Proportion of NSCLC patients who receive EGFR test before combination therapy | NSCLC patients who receive EGFR test before combination therapy | NSCLC patients staging Ⅳ who accept combination therapy |
| Proportion of pathology report available in the chart for NSCLC patients who have surgical resection | NSCLC patients whose pathology report available are in the chart | NSCLC patients who have surgical resection |
| Proportion of NSCLC patients who obtain FEV1 and DLCO within 2 weeks before lung resection | NSCLC patients who obtain FEV1 and DLCO within 2 weeks before lung resection | NSCLC patients who have surgical resection |
| Proportion of NSCLC patients who receive ECG within 2 weeks before lung resection | NSCLC patients who receive ECG within 2 weeks before lung resection | NSCLC patients who have surgical resection |
| Proportion of NSCLC patients staging I or II without contraindications who undergo curative resection | NSCLC patients who undergo curative resection | NSCLC patients staging I or II without contraindications |
| Proportion of NSCLC patients staging IA without contraindications who undergo lobectomy | NSCLC patients who undergo lobectomy | NSCLC patients staging IA without contraindications |
| Proportion of NSCLC patients staging IB or II who receive lobectomy with adjuvant chemotherapy or lobectomy only | NSCLC patients who undergo lobectomy with adjuvant chemotherapy or lobectomy only | NSCLC patients staging IB or II |
| Proportion of NSCLC patients with stage IIA, IIB or Ⅲ A who receive adjuvant chemotherapy after curative resection | NSCLC patients who receive adjuvant chemotherapy | NSCLC patients with stage IIA, IIB or ⅢA who receive curative resection |
| Proportion of NSCLC patients with stage IIA, IIB or Ⅲ A who receive cisplatin-based adjuvant chemotherapy within 3 to 4 weeks after undergoing curative resection | NSCLC patients who receive cisplatin-based adjuvant chemotherapy within 3 to 4 weeks after curative resection | NSCLC patients with stage IIA, IIB or ⅢA |
| Proportion of NSCLC patients staging ⅢB with malignant effusion or Ⅳ who receive first-line chemotherapy | NSCLC patients who receive first-line chemotherapy | NSCLC patients staging ⅢB with malignant pleural effusion or Ⅳ with normal organ function and good performance status |
| Proportion of NSCLC patients staging ⅢB or Ⅳ who receive imaging study to assess response of chemotherapy at least once before the completion of four cycles | NSCLC patients who receive imaging study to assess response of chemotherapy at least once before the completion of four cycles | Proportion of NSCLC patients staging ⅢB with malignant pleural effusion or Ⅳ who receive chemotherapy |
| Proportion of NSCLC patients staging I or II pathologically who receive postoperative radiation therapy after incomplete surgical resection | NSCLC patients who receive postoperative radiation therapy | NSCLC patients staging I or II pathologically who undergo incomplete surgical resection |
| Proportion of locally advanced NSCLC patients who receive neo-adjuvant chemotherapy | NSCLC patients who receive neo-adjuvant chemotherapy | Locally advanced NSCLC patients |
| Proportion of locally advanced NSCLC patients with performance status 0 or 1 who receive combination therapy | NSCLC patients who receive combination therapy | Locally advanced NSCLC patients with performance status 0 or 1 |
| **Communication indicators** |  |  |
| Proportion of NSCLC patients who are informed of a follow-up plan at the time of discharge from hospital | NSCLC patients who is informed of a follow-up plan at the time of discharge from hospital | All the NSCLC patients |
| Proportion of active smokers with NSCLC who have had smoking cessation counseling documented | NSCLC patients who have had smoking cessation counseling documented | Active smokers with NSCLC |
| Proportion of NSCLC patients staging IA who are recommend adjuvant chemotherapy after curative resection (lower score: better) | NSCLC patients who are recommended adjuvant chemotherapy | NSCLC patients staging IA who undergo curative resection |
| **Outcome indicators** |  |  |
| Proportion of NSCLC patients who have post-operative complications | NSCLC patients who have postoperative complications | NSCLC patients who undergo surgery |

**References**

1. Birkmeyer JD, Sun Y, Goldfaden A, Birkmeyer NJ, Stukel TA: **Volume and process of care in high-risk cancer surgery**. *Cancer* 2006, **106**(11):2476-2481.

2. Birkmeyer NJ, Goodney PP, Stukel TA, Hillner BE, Birkmeyer JD: **Do cancer centers designated by the National Cancer Institute have better surgical outcomes?** *Cancer* 2005, **103**(3):435-441.

3. Cheung MC, Hamilton K, Sherman R, Byrne MM, Nguyen DM, Franceschi D, Koniaris LG: **Impact of Teaching Facility Status and High-Volume Centers on Outcomes for Lung Cancer Resection: An Examination of 13,469 Surgical Patients**. *Annals of Surgical Oncology* 2009, **16**(1):3-13.

4. **The National Quality Forum.** Available at: <http://www.qualityforum.org/QPS/0381> Accessed July 4 2013.

5. Hermens RPMG, Ouwens MMTJ, Vonk-Okhuijsen SY, Wel YVD, Tjan-Heijnen VCG, Broek LDVD, Ho VKY, Janssen-Heijnen MLG, Groen HJM, Grol RPTM: **Development of quality indicators for diagnosis and treatment of patients with non-small cell lung cancer: A first step toward implementing a multidisciplinary, evidence-based guideline**. *Lung cancer (Amsterdam, Netherlands)* 2006, **54**(1):117-124.

6. Ouwens MM, Hermens RR, Termeer RA, Vonk-Okhuijsen SY, Tjan-Heijnen VC, Verhagen AF, Hulscher MM, Marres HA, Wollersheim HC, Grol RP: **Quality of integrated care for patients with nonsmall cell lung cancer: variations and determinants of care**. *Cancer* 2007, **110**(8):1782-1790.

7. Ouwens M, Hermens R, Hulscher M, Vonkokhuijsen S, Tjanheijnen V, Termeer R, Marres H, Wollersheim H, Grol R: **Development of indicators for patient-centred cancer care**. *Supportive Care in Cancer* 2010, **18**(1):121-130.

8. Tanvetyanon T: **Quality-of-care indicators for non-small cell lung cancer**. *Cancer Control Journal of the Moffitt Cancer Center* 2009, **16**(4):335-341.

9. **National Quality Measures Clearinghouse.** Available at:<http://www.qualitymeasures.ahrq.gov/> Accessed July 4 2013.

10. Cassivi SD, Allen MS, Vanderwaerdt GD, Ewoldt LL, Cordes ME, Wigle DA, Nichols FC, Pairolero PC, Deschamps C: **Patient-Centered Quality Indicators for Pulmonary Resection**. *Annals of Thoracic Surgery* 2008, **86**(3):927-932.

11. Ettinger DS, Akerley W, Borghaei H, Chang AC, Cheney RT, Chirieac LR, D'Amico TA, Demmy TL, Ganti AKP, Govindan R: **NCCN Clinical Practice Guidelines in Oncology™ Non-Small Cell Lung Cancer**. *Journal of the National Comprehensive Cancer Network Jnccn* 2012, **10**.

12. Oxnard GR, Fidias P, Muzikansky A, Sequist LV: **Non-small cell lung cancer in octogenarians: treatment practices and preferences**. *Journal of Thoracic Oncology Official Publication of the International Association for the Study of Lung Cancer* 2007, **2**(11):1029-1035.

13. Potosky AL, Saxman S, Wallace RB, Lynch CF: **Population Variations in the Initial Treatment of Non–Small-Cell Lung Cancer**. *Journal of Clinical Oncology Official Journal of the American Society of Clinical Oncology* 2004, **22**(16):3261-3268.

14. Blayney DW, Miela G, Markstrom D, Hanauer D, Mcniff K, Neuss M: **Measuring quality with the Quality Oncology Practice Initiative (QOPI) at a university comprehensive cancer center**. *Journal of Clinical Oncology* 2007.

15. **ASCO QOPI Summary Measures Fall 2012.** Available at: <http://qopi.asco.org> Accessed July 4 2013.

16. Chien CR, Tsai CM, Tang ST, Chung KP, Chiu CH, Lai MS: **Quality of care for lung cancer in Taiwan: a pattern of care based on core measures in the Taiwan Cancer Database registry**. *Journal of the Formosan Medical Association* 2008, **107**(8):635-643.

17. Chien CR, Lai MS: **Trends in the Pattern of Care for Lung Cancer and Their Correlation With New Clinical Evidence: Experiences in a University-Affiliated Medical Center**. *American Journal of Medical Quality* 2006, **21**(6):408-414.

18. Meert AP, Martin B, Delmotte P, Berghmans T, Lafitte JJ, Mascaux C, Paesmans M, Steels E, Verdebout JM, Sculier JP: **The role of EGF-R expression on patient survival in lung cancer: a systematic review with meta-analysis**. *European Respiratory Journal* 2002, **20**(4):975-981.

19. Herbst RS, Maddox AM, Rothenberg ML, Small EJ, Rubin EH, Baselga J, Rojo F, Hong WK, Swaisland H, Averbuch SD *et al*: **Selective oral epidermal growth factor receptor tyrosine kinase inhibitor ZD1839 is generally well-tolerated and has activity in non-small-cell lung cancer and other solid tumors: results of a phase I trial**. *Journal of clinical oncology : official journal of the American Society of Clinical Oncology* 2002, **20**(18):3815-3825.

20. **M. D. Anderson Physicians Network Provider News. Quality Indicators for Concordance Study.** Available at: <http://www.mdanderson.org/education-and-research/resources-for-professionals/clinical-tools-and-resources/physicians-network/physicians-network.html> Accessed July 4 2013.

21. Jan M, Anne-Marie H, Torben P, Bartels PD: **National quality measurement using clinical indicators: the Danish National Indicator Project**. *Journal of Surgical Oncology* 2009, **99**(8):500–504.

22. Mainz J, Hjulsager M, Og MTE, Burgaard J: **National benchmarking between the Nordic countries on the quality of care**. *Journal of Surgical Oncology* 2009, **99**(8):505-507.
